# Adopting a smart toothbrush with artificial intelligence may improve oral care in patients admitted to the intensive care unit

**DOI:** 10.1186/s13054-019-2512-8

**Published:** 2019-06-18

**Authors:** Tommaso Scquizzato, Arianna Gazzato

**Affiliations:** 1 Department of Anesthesia and Intensive Care, IRCSS San Raffaele Scientific Institute, Milan, Italy; 2 Vita-Salute San Raffaele University, Milan, Italy; 3 Azienda ULSS 3 Serenissima - Ospedale di Mirano, Venice, Italy

Dear Editor,

Ventilator-associated pneumonia (VAP) is one of the most common complications in the intensive care unit (ICU) [1]. VAP, developed in patients mechanically ventilated for at least 48 h, increases time on mechanical ventilation, length of ICU stay, and mortality [2, 3].

Providing a systematic and effective oral hygiene is crucial in the ICU. Lack of this aspect of care favors an increase of dental plaque deposits on the teeth becoming potential reservoirs for bacteria that could progress to the respiratory system causing pneumonia [4, 5].

The aim of oral hygiene is to regularly remove the plaques from the teeth especially along the gingival margin and proximal tooth surface to prevent aspiration of oropharyngeal colonization, one major factor causing VAP. Teeth should be regularly cleaned using a mouth rinse, toothbrush, or combination of both, together with suctioning secretions.

Electronic toothbrushes released in markets in the last years are improving the way people brush their teeth. These smart toothbrushes embedded with built-in 3D sensors collect and analyze data about tooth brushing accuracy, duration, and frequency detecting real-time position and orientation across different brushing zones. Thanks to the artificial intelligence (AI) and machine learning algorithms, the device keeps track of the skipped areas and offers custom recommendations to improve oral care. The missed areas are generally indicated in a tooth map reporting also the percentage of brushed surface, the teeth that require more attention, and if the user is applying too much pressure in a particular area that may provoke gingival damage.

By taking advantage of AI-powered analysis, ICU staff in charge for oral care of patients might optimize and improve their technique, complete brushing of missed surfaces, and adjust the pressure used obtaining a total and accurate mouth cleaning without missing a tooth.

Integrating smart toothbrushes in an oral hygiene program might improve oral care and prevent serious complications such as pneumonia in critically ill patients admitted to the ICU. Some aspects that deserve to be further investigated in order to understand the actual implementability of these devices in the ICUs are toothbrush sterilization, the availability of disposable parts, and the increased price in respect to standard toothbrushes. These devices usually have the possibility to replace brush heads while the body is fixed and the possibility to sterilize it is unknown.

## Data Availability

Not applicable.

## Abbreviations

AI: Artificial intelligence
ICU: Intensive care unit
VAP: Ventilator-associated pneumonia
